# A Novel Neuraminidase Virus-Like Particle Vaccine Offers Protection Against Heterologous H3N2 Influenza Virus Infection in the Porcine Model

**DOI:** 10.3389/fimmu.2022.915364

**Published:** 2022-07-07

**Authors:** Vasilis C. Pliasas, Zach Menne, Virginia Aida, Ji-Hang Yin, Maria C. Naskou, Peter J. Neasham, J. Fletcher North, Dylan Wilson, Katharine A. Horzmann, Joshy Jacob, Ioanna Skountzou, Constantinos S. Kyriakis

**Affiliations:** ^1^ Department of Pathobiology, College of Veterinary Medicine, Auburn University, Auburn, AL, United States; ^2^ Emory-UGA Center of Excellence for Influenza Research and Surveillance (CEIRS), Atlanta, GA, United States; ^3^ Department of Microbiology and Immunology, School of Medicine, Emory University, Atlanta, GA, United States; ^4^ Center for Vaccines and Immunology, University of Georgia, Athens, GA, United States

**Keywords:** neuraminidase, influenza A virus, virus like particles, vaccine, swine

## Abstract

Influenza A viruses (IAVs) pose a global health threat, contributing to hundreds of thousands of deaths and millions of hospitalizations annually. The two major surface glycoproteins of IAVs, hemagglutinin (HA) and neuraminidase (NA), are important antigens in eliciting neutralizing antibodies and protection against disease. However, NA is generally ignored in the formulation and development of influenza vaccines. In this study, we evaluate the immunogenicity and efficacy against challenge of a novel NA virus-like particles (VLPs) vaccine in the porcine model. We developed an NA2 VLP vaccine containing the NA protein from A/Perth/16/2009 (H3N2) and the matrix 1 (M1) protein from A/MI/73/2015, formulated with a water-in-oil-in-water adjuvant. Responses to NA2 VLPs were compared to a commercial adjuvanted quadrivalent whole inactivated virus (QWIV) swine IAV vaccine. Animals were prime boost vaccinated 21 days apart and challenged four weeks later with an H3N2 swine IAV field isolate, A/swine/NC/KH1552516/2016. Pigs vaccinated with the commercial QWIV vaccine demonstrated high hemagglutination inhibition (HAI) titers but very weak anti-NA antibody titers and subsequently undetectable NA inhibition (NAI) titers. Conversely, NA2 VLP vaccinated pigs demonstrated undetectable HAI titers but high anti-NA antibody titers and NAI titers. Post-challenge, NA2 VLPs and the commercial QWIV vaccine showed similar reductions in virus replication, pulmonary neutrophilic infiltration, and lung inflammation compared to unvaccinated controls. These data suggest that anti-NA immunity following NA2 VLP vaccination offers comparable protection to QWIV swine IAV vaccines inducing primarily anti-HA responses.

## Introduction

Influenza A viruses (IAV) constitute a major public health concern and their pandemic potential highlights the need for vaccines that induce broadly protective immune responses against potential novel zoonotic IAV strains (1). Currently available seasonal human influenza vaccines are standardized by hemagglutinin (HA) content only and, therefore, induce primarily anti-HA immune responses (2). Available influenza vaccines have historically demonstrated sub-optimal efficacy, especially against heterologous IAV viruses and newly emerged novel IAVs (3, 4). The immunodominance of HA observed after vaccination has led to selection of escape mutants that result in antigenic mismatch between the HA in seasonal vaccines and the HA in circulating viruses at the time of infection, which contributes to the observed low vaccine effectiveness of current seasonal vaccines (5).

Neuraminidase (NA) is the second most abundant glycoprotein on the surface of influenza virions and exists as a homotetramer typically present at a ratio of 1 to 4-5 with respect to HA (6). NA functions as a sialidase, cleaving terminal sialic acid residues from both host and viral glycosylated proteins to enable virion motility to receptor-dense regions of the epithelium and progression to the lower respiratory tract (7, 8). In addition to promoting viral uptake and infectivity, enzymatic removal of sialic acids prevents virion aggregation at the surface of infected cells, facilitating the efficient detachment and spread of nascent progeny virions (8, 9). In the quest for improved influenza vaccines and conserved immunogenic epitopes which can induce broadly reactive protection, neuraminidase has been identified as a candidate for use as a vaccine antigen that can potentially elicit broadly reactive NA-specific protective immune responses against heterologous viruses (2, 10, 11).

Contrary to HA specific immunity where the majority of HA-induced antibodies prevent viral infection by blocking HA receptor-binding or fusion activity, humoral responses directed against NA do not elicit a neutralizing immune response. Rather, anti-NA antibodies are characterized by inducing infection-permissive immunity (12, 13). Rather than preventing viral infection, antibodies targeting neuraminidase inhibit nascent virion release through the inhibition of enzymatic activity by sterically blocking access of substrates to the NA catalytic pocket and preventing cleavage (14). In effect, these neuraminidase inhibiting antibodies limit viral replication and dissemination within the host by tethering newly budded virion clumps to the infected cell plasma membrane (9, 15). In addition to inhibiting NA enzymatic activity, anti-NA antibodies have also been reported to facilitate viral clearance by mediating recognition and killing of infected cells by immune effector cells through antibody-dependent cell-mediated cytotoxicity (ADCC) (16). However, anti-NA immunity has also been shown to be protective in Fc-gamma receptor knock-out mice, suggesting ADCC effector functions are not necessary for NA mediated protection in murine models of IAV infection (17).

A number of vaccination-challenge studies have demonstrated that NA immunization using different types of vaccine platforms has conferred sufficient protective immunity to reduce both disease morbidity and mortality while limiting virus shedding (18–20). As a platform, the use of virus like particles (VLPs) is an attractive option for NA presentation due to their resemblance in morphology, size, and structural conformation of viruses as well as their ability to present antigen in its native, membrane bound conformation (21). In addition to their immunogenicity, VLPs offer advantages with respect to safety as they are void of genetic material making VLPs non-replicating molecules without the risk of reversion to virulence. Additionally, VLP vaccine development is cost effective and offers the opportunity for a customizable vaccine approach allowing for the selective inclusion of specific antigens (22). Previous reports of vaccination with HA VLP constructs demonstrated protective immune responses against homologous and heterologous seasonal influenza viruses as well as against potentially pandemic influenza viruses (23–25).

Recent studies have reported that intranasal and intramuscular vaccination of mice with NA1 VLPs produced in insect cells induce heterosubtypic immunity in a lethal murine infection model (26). An initial study demonstrated that intranasal immunization of mice with VLPs containing the NA1 and matrix 1 (M1) protein from the H1N1 PR8 strain was protective against a lethal challenge with the H3N2 A/Philippines/82 influenza strain. A subsequent study by the same investigators reported that the NA and M1 proteins from the 2009 pandemic H1N1 strain (H1N1pdm09) conferred protection against lethal challenge with a A/Philippines/28 and a recombinant virus containing the HA and NA genes from the highly pathogenic avian influenza H5N1 A/Vietnam/1203/2004 strain, resulting in reduced pulmonary viral titers and reduced pathology. However, investigations by our group and others using NA VLPs produced in insect cell expression systems have failed to show protection against heterosubtypic challenge (27, 28).

Of the 9 subtypes of IAV neuraminidase (NA1-NA9) that have been identified in influenza viruses circulating in wild waterfowl, only the NA1 and NA2 subtypes currently circulate in human and swine populations (29). Pigs are natural hosts of influenza A viruses and have comparable distribution of sialic acid receptors to humans throughout their respiratory tract (30). These shared receptor distributions enable influenza A viruses to cross species barriers, adapt, and establish endemic transmission in new populations resulting in the co-circulation of H1N1 and H3N2 subtypes in both humans and swine (31). Additionally, similar clinical manifestations, pathogenesis, and immunological responses to influenza infection and vaccination in humans and pigs make the latter an ideal animal model for the study of vaccine immunogenicity, efficacy, and influenza A virus pathogenesis (32–34).

Currently, no studies investigating the immunogenicity and breadth of protection of NA2 neuraminidase in large animal models have been reported. Investigation of anti-NA2 immunity in additional animal models is warranted as H3N2 influenza viruses have recently demonstrated increased pathogenicity compared to H1N1 viruses, resulting in higher rates of morbidity and mortality in the elderly and other at risk populations (35). While previous studies have demonstrated that NA inhibitory (NI) antibody titers are associated with the induction of protective immunity in pigs, the full extent of anti-NA immunity, including humoral and cell-mediated immune responses, has not been properly investigated in the swine model (36–38). Furthermore, the immunogenicity of NA VLPs has yet to be explored in the swine model.

In the current study, we evaluated neuraminidase 2 virus-like particles (NA2 VLPs) as a candidate influenza vaccine in a vaccination-challenge model in swine. Specifically, we investigated the immunogenicity of NA2 (A/Perth/16/2009 H3N2 strain) and M1 (A/Michigan/73/2015 H1N1 strain) VLP formulation in swine and efficacy after challenge with the heterologous H3N2 swine field isolate A/swine/NC/KH1552516/2016. This work demonstrates the immunogenicity and efficacy of NA2 VLPs in a swine model and highlights the contribution of anti-NA immunity in contrast to the anti-HA immunity provided by currently available swine vaccines.

## Materials and Methods

### Cells and Virus Stocks

*Spodoptera frugiperda* (Sf9) cells (IPLB-Sf-21-AE, Expression Systems, Davis, CA, USA) were maintained at 27˚C in suspension in shaker flasks using serum free SF900II media (Gibco/ThermoFisher Scientific, Waltham, MA, USA). *Trichoplusia ni* (Tni) cells (Expression Systems, Davis, CA, USA) were maintained at 27˚C in suspension in shaker flasks using serum free ESF 921 media (Expression Systems, Davis, CA, USA). Madin-Darby canine kidney (MDCK) cells (ATCC CCL 34, American Type Culture Collection, Manassas, VA, USA) were maintained in Dulbecco’s modified Eagle’s media (DMEM) (Corning Life Sciences, Corning, NY, USA) supplemented with 10% fetal bovine serum (GE Healthcare Life Sciences, Westborough, MA, USA), 1% antibiotic-antimycotic solution (Corning Life Sciences, Corning, NY, USA), in a 37°C incubator with 5% CO_2._ Animals were challenged with A/swine/NC/KH1552516/2016, a Cluster IV-A H3N2 virus isolated from a recent outbreak of respiratory disease in a swine herd (39). Its NA protein shared a 90.9% amino acid homology with the human NA of A/Perth/16/2009 strain, included in the NA VLP vaccine. The virus was propagated in MDCK cells, and the challenge titer was determined by 50% median tissue culture infectious dose (TCID_50_) calculated using the Reed and Muench formula (40). Challenge virus stocks were aliquoted for single-use applications and stored at −80°C. On the day of challenge, virus was prepared for an inoculum of 10^6^ TCID_50_/ml. Inoculum was administered intranasally in 2 ml total volume (1 ml per nostril, total challenge dose of 10^6.3^ TCID_50_) using a mucosal atomization device (MAD Nasal™ Atomization Device, Teleflex, Wayne, PA).

### Animals

Eighteen 6-week-old influenza-naive conventional Yorkshire/Hampshire cross pigs, were obtained from Auburn University’s Swine Research and Education Center (influenza virus and porcine reproductive and respiratory syndrome virus-seronegative farm). All study procedures were reviewed and approved by the Institutional Animal Care and Use Committee (IACUC) of Auburn University. Until one week prior to challenge, animals were housed in industry-standard pens in groups of 10 at the Swine Research and Education Center and were provided food and water ad libitum. All eighteen pigs arrived at the age of 13 weeks into the BSL-2 facilities of the Sugg Laboratory for Animal Health Research. Pigs were randomly divided in four groups as outlined in the swine vaccination and virus challenge section. Each experimental group was housed in a separate isolation unit with HEPA filtered air. Food and water were provided *ad libitum*.

### VLP Production and Purification

Neuraminidase 2 virus like particles (NA2 VLPs) were produced and purified as previously described (27). Briefly, sequence information for the NA2 gene from A/Perth/16/2009 and the M1 gene from A/Michigan/73/2015 were synthesized with the addition of a unique BamHI endonuclease restriction site at the 5’ end and a unique HindIII endonuclease restriction site at the 3’ end (Integrated DNA technologies, Coralville, IA, USA). Gene fragments were digested and directionally cloned into a pFastBac1 vector (ThermoFisher Scientific, Waltham, MA, USA). Baculovirus stocks were created according to the Bac-to-Bac^®^ manufacturer’s instructions (ThermoFisher Scientific, Waltham, MA, USA). The resulting baculovirus stocks infected Tni cells at a multiplicity of infection (MOI) of 5 for NA2 expressing recombinant baculoviruses (rBVs) and 3 for M1 rBVs. VLPs were purified from the culture supernatant as previously described (27).

### Vaccine Preparation

NA2 VLP antigen was co-formulated with the Water-in-Oil-in-Water (W/O/W) emulsion (Montanide™ ISA 206 VG) which served as the adjuvant. Vaccine formulation was prepared under low shear rate and stored according to the manufacturer’s instructions, one day prior each vaccination. For the commercial vaccine, we used FluSure XP^®^ (Zoetis Inc., Kalamazoo, MI, USA) which is a licensed swine adjuvanted (Amphigen®) quadrivalent whole inactivated virus (QWIV) influenza vaccine, that contains H1N1 Gamma cluster (virus) and H3N2 Cluster IV strains, A/swine/NC/394/2012, which is a Cluster IV-A virus and A/swine/MN872/2012, which is a Cluster IV-B virus, and an H1N2 Delta-1 virus. The amino acid homology between the HA of A/swine/NC/394/2012, included in the QWIV vaccine and the HA of the challenge virus was 97.78%. Animals were vaccinated with either the recombinant NA2 VLP vaccine, the FluSure XP^®^ commercial vaccine or a Phosphate-Buffered Saline (PBS) in adjuvant solution. Each pig received a deep intramuscular injection into the neck with 2 ml of the FluSure XP^®^ commercial vaccine, 1 ml of the NA2 VLPs vaccine (60 μg/mL) or 1 ml of phosphate-buffered saline with W/O/W adjuvant.

### Swine Vaccination, Virus Challenge, and Sample Collection

Pigs were randomly allocated to one of three groups. Group 1 pigs (n = 6) were vaccinated with the NA2 VLP vaccine co-formulated with the water-in-oil-in-water (W/O/W) adjuvant (ISA 206, Seppic). Group 2 pigs (n = 6) were vaccinated with the QWIV vaccine (FluSure XP, Zoetis). Group 3 pigs (n=6) were mock vaccinated with W/O/W adjuvant (ISA 206, Seppic) only and served as controls. Prime immunization of the animals was conducted at the age of 6 weeks and booster immunization was administered 3 weeks later, at the age of 9 weeks. Throughout the course of the immunogenicity stage, the animals were housed at the Swine Research and Education Center.

Three weeks after the administration of the boost vaccination and one week prior to the onset of the animal challenge, all eighteen pigs were transferred into the BSL-2 facilities of the Sugg Laboratory for Animal Health Research. They were randomly divided into four groups: two groups of 6 pigs, including 2 NA2 VLP vaccinated animals, 2 QWIV vaccinated animals and 2 mock vaccinated controls; the other two groups were comprised of 3 animals; 1 NA2 VLP vaccinated animal, 1 QWIV vaccinated pig and 1 mock vaccinated control. The four groups of animals were housed in separate isolation units and were provided food and fresh water *ad libitum*. Serum was collected prior to prime and boost vaccination, at 2 weeks post-boost, and at 4 weeks post-boost prior to challenge. Four weeks after boost, the two groups of 6 and one group of 3 animals (n=15), were challenged with A/swine/NC/KH1552516/2016 (10^6.3^ TCID_50_ in 2 mL of PBS) by intranasal inoculation using a mucosal atomization device (MAD Nasal™ Atomization Device, Teleflex, Wayne, PA). The fourth group of 3 animals was not challenged and served as the non-infected control. However, one pig of the fourth group was removed from the study due to a medical issue unrelated to the experiment. Animals were clinically scored daily post-infection based on rectal temperature, respiratory rate, and assessment of clinical demeanor. Specifically, rectal temperature score ranged from 0 to 3 (< 39.4°C = 0, 39.4-39.9°C = 1, 40-40.5°C = 2, > 40.6°C = 3), respiratory rate per minute score ranged from 0 to 2 (20-40 = 0, 41-59 = 1, > 60 = 2) and clinical behavior score, based on coughing (absent = 0, present = 1) and depression (absent = 0, present = 2) ranged from 0 to 3. Nasal swabs were collected daily starting two days prior to challenge until day 5, for the valuation of viral shedding. All animals were humanely euthanized with a lethal dose of pentobarbital on day 5 post-infection. On the day of euthanasia, bronchoalveolar lavage fluid (BALF) was harvested for cytopathology and pigs were necropsied. During necropsy, nasal turbinates, trachea, right lung lobes (including accessory lobe) and left lung lobes were collected for histopathological evaluation and examination of viral replication.

### Measurement of NA-Specific IgG Antibody Titers

Sera from individual animals were evaluated for end-point dilution antibody titers as previously described with modifications (41). Briefly, Nunc Maxisorb 96-well plates (ThermoFisher Scientific, Waltham, MA, USA) were coated overnight at 4˚C with 100 ng/well of purified recombinant N2 protein (rN2) produced using the sequence information from A/Wisconsin/67/2005 (BEI Resources #NR-19237, Manassas, VA, USA). After overnight coating, plates were blocked for 1 hour at room temperature with 200 µl of blocking solution (1x PBS with 0.1% Tween 20) followed by an automated wash step (4x with 1x PBS-T, 1x PBS with 0.1% Tween 20). Sera were then serially diluted and incubated as previously described followed by the same wash step. Plates were then incubated with goat anti-porcine IgG secondary antibody (Novus Biologicals, Centennial, CO, USA) followed by an additional wash step. Plates were then incubated with rabbit anti-goat-HRP detection antibody (Southern Biotech, Birmingham, AL, USA) followed by an additional wash step. Plates were developed for 20 minutes at room temperature after addition of OPD substrate (ThermoFisher Scientific, Waltham, MA, USA). Plate absorbance was read at 450 nm using an xMark Microplate Spectrophotometer (BioRad, Hercules, CA, USA). The end point dilution titer of a sample was defined as the highest reciprocal serum dilution where the optical density (OD) measured greater than two times the mean of the blank sample.

### Neuraminidase Activity and Inhibition Assay

Purified VLPs preparations were tested for enzymatic neuraminidase activity using the NA-Star Influenza Neuraminidase Inhibitor Resistance Detection Kit (ThermoFisher Scientific, Waltham, MA, USA) according to the manufacturer’s instructions. Briefly, VLP solutions were diluted in NA-Star Assay Buffer and then incubated with substrate for 30 min at room temperature. NA-Star accelerator solution was then injected followed by measurement of the chemiluminescent signal in relative luminescent units (RLU) *via* Modulus II Microplate Luminometer (Promega, Madison, WI, USA). Inhibition of neuraminidase activity was measured similarly and as previously described with modifications (42). Briefly, 25 µl of processed sera diluted in NA-Star buffer was incubated with 40 ng of NA2 VLPs diluted in NA-Star buffer or 1.04x10^4^ TCID_50_ of A/swine/NC/KH1552516/2016 diluted in NA-Star buffer in a volume of 25 µl for a total volume of 50 µl at 37˚C for 20 minutes. Diluted NA-Star Substrate was added in a volume of 10 µl and incubated at room temperature for 30 minutes. The chemiluminescent reaction was initiated upon injection of 60 µl of NA-Star Accelerator followed by measurement using the Modulus II Microplate Luminometer (Promega, Madison, WI, USA). The reciprocal of the highest serum dilution resulting in an inhibition of 50% of NA activity compared to NA-Star buffer alone was defined as the NAI titer.

### Hemagglutination Inhibition Assay

Sera for hemagglutination inhibition titers were processed according to the WHO protocol (43). Serum samples were treated for 1 hour at 37°C with receptor destroying enzyme (RDE) (Denka Seiken Co Ltd) to remove nonspecific inhibitors. The residual RDE and the complement were inactivated at 56°C for 30 minutes. Sera were incubated with packed turkey RBC overnight at 4°C, for removal of non-specific cryoglobulins interfering with RBC and antigen-antibody binding. Two-fold serially diluted sera were co-incubated with 4 HA units of MDCK grown A/swine/NC/KH1552516/2016 H3N2 stock for one hour followed by the addition of 0.5% turkey red blood cells for 45 minutes, all steps performed at room temperature (44). The HAI titers were read as the reciprocal of the highest dilution of serum that conferred inhibition of hemagglutination.

### Cytopathological Evaluation of Bronchoalveolar Lavage Fluid

After euthanasia, lungs were collected from all the (n=17) for tissue and bronchoalveolar lavage fluid (BALF) harvesting. The lungs were washed with Ca/Mg deficient PBS (Corning Life Sciences, Corning, NY, USA). Following BALF collection, samples were filtered through a cell strainer (70 µm pore size) (VWR International, Radnor, PA) to remove mucous and centrifuged at 1200 x g for 5 minutes. After centrifugation, the supernatant was collected and stored in aliquots at -80°C for further use. Before use, the pelleted cells were resuspended in 1X Lysis Buffer (BioLegend, San Diego, CA) and incubated for 5 minutes at room temperature. The cell suspension was then quenched with an equal volume of PBS and centrifuged at 1200 x g for 5 minutes. After supernatant was discarded, BAL cells were counted (Countess II Automated Cell Counter, Thermo Fisher Scientific, Carlsbad, CA) and resuspended with the appropriate volume of PBS to achieve a final density of 0.5-1 x 10^5^ cells, added to the slide and cytocentrifuged at 1200 rpm for 5 minutes. The cytocentrifuged smear was stained with a three step Wright-Giemsa Stain (Quick Stain, VWR International, Radnor, PA) for cytological evaluation. A differential cell count was determined microscopically by evaluating 300 cells on a cytospin smear. Epithelial cells were not included in the differential count and the cell differentials in BAL fluid were expressed as the percentage of the total number of cells counted (45).

### Virus Titration (TCID_50_) Assay

Virus titration assays were performed on nasal swabs and respiratory tissue homogenates to evaluate viral shedding and virus replication between vaccinated and unvaccinated animals. Nasal swabs collected daily post-challenge from each animal were first suspended with 2ml of phosphate-buffered saline (PBS) supplemented with 1% antibiotic-antimycotic solution, then mechanically agitated for 10 minutes at 4°C and stored as aliquots at -80°C for further use in virus titration assays. Additionally, respiratory tissue samples (nasal turbinates, trachea, right, left and accessory lung lobes) harvested after euthanasia were also processed prior to virological evaluation. Tissue samples were weighed and then suspended with cold PBS supplemented with 2% antibiotic-antimycotic solution cocktail to reach 10% gr of tissue dissolved in PBS. Tissue suspensions were then homogenized and centrifuged. Subsequently, tissue homogenate supernatants were collected, aliquoted and stored at -80°C for TCID_50_ assays.

Tested samples were initially serially tenfold diluted and transferred to confluent MDCK cell cultures in 96-flat bottomed well plates and were subsequently incubated at 37°C for 72 hours. After 72 hours, wells were subjected to microscopic evaluation for the presence of CPE and virus titers were determined by the Reed-Muench method (40).

### Quantification of Cytokine Secreting Cells

Peripheral Blood Mononuclear Cells (PBMCs) were collected from the 15 animals that were challenge at: (a) 3 weeks post-prime vaccination (day 21) and (b) 2 weeks post-boost vaccination (day 35), as previously described (46). Cryopreserved PBMC aliquots were thawed, washed with RMPI medium (Hyclone Laboratories Inc., Logan, UT) and resuspended in complete RPMI medium (10% FBS, 1% penicillin/streptomycin, Hepes buffer and 0.1% beta-mercaptoethanol) for ELISPOT assays. The numbers of IAV-specific Interferon-γ (IFN-γ-STc) and TNF-α secreting T-cells (TNF-α-STc) were determined with swine IFN-γ and TNF-α ELISpot BASIC kit (Mabtech Inc., Cincinnati, OH), following the manufacturer’s instructions with minor adjustments (47). Briefly, for IFNγ ELISpot assay, 2x10^5^ cells/well from swine vaccinated with FluSure, NA2 VLPs or mock vaccinated (adjuvant alone) were stimulated with either 10^6^ TCID of the challenge virus (A/swine/NC/KH1552516/2016), 0.5 μg/ml PHA (ThermoFisher Scientific, Waltham, MA, USA) or with medium alone for 48 hours at 37°C. Regarding TNF-α ELISpot assay, 10^5^ cells/well from swine vaccinated with QWIV, NA2 VLPs or mock vaccinated (adjuvant alone) were stimulated with either 10^4^ TCID of the challenge virus (A/swine/NC/KH1552516/2016), 1 μg/ml PHA or with medium alone for 48 hours at 37°C. After incubation, the plates were washed with PBS and incubated with biotin labeled detection antibody (0.5μg/ml) for 2 hours. Following the addition of streptavidin-horseradish peroxidase for 1 hour, TMB substrate solution (Mabtech Inc., Cincinnati, OH) was then used for spot development until detection of distinct spots.

### Quantification of Cytokine Secretion in Lung Homogenates

Cytokines were quantified in pulmonary lung homogenates from swine vaccinated with QWIV, NA2 VLPs or mock vaccinated (adjuvant alone) collected at day 5 post-infection, using the 13 analyte (GM-CSF, IFN-γ, IL-1α, IL-1ra, IL-1β, IL-2, IL4, IL-6, IL-8, IL-10, IL-12, IL-18 and TNF-α) MILLIPLEX MAP Porcine Cytokine/Chemokine Magnetic Bead Panel per manufacturer’s instructions (EMD Millipore Corporation, Billerica, MA). Lung TNF-a was quantified using a Porcine TNF-a ELISA kit (R&D Systems Inc., Minneapolis, MN) according to manufacturer’s instructions.

### Macroscopic and Microscopic Evaluation of Respiratory Tissues

At necropsy, macroscopic scoring of lung lesions was performed to estimate the percentage of lung lobes affected (48). Dark purple, depressed, firm areas of the lung lobes were considered a typical lesion for swine influenza virus infection. Additionally, sections of nasal turbinates, trachea and all seven lung lobes were collected from each individual pig for histopathological evaluation and were placed in 10% neutral buffered formalin for fixation. Tissues were routinely processed, embedded in paraffin, cut at 5 µm, stained with H&E, and examined by light microscopy (Olympus BX53; Olympus Scientific Solutions Technologies Inc., Waltham, MA). A modified version of a swine influenza grading system was applied to obtain histopathologic scores (49). Histopathology scores considered the percent of lung affected, extent of bronchial and bronchiolar epithelial changes, degree of lymphocytic peribronchiolar cuffing, and the severity of interstitial pneumonia. Each criterion was scored from 0 to 4, with 4 being most severe, and added to obtain an overall score. Trachea was evaluated for epithelial changes and degree of tracheitis and was scored from 0 to 3. For the nasal turbinates, epithelial changes and the degree of rhinitis were examined and scored 0 to 3. Blinded microscopic evaluation was performed by two individuals, including a board-certified veterinary pathologist and the scores were averaged.

### Statistical Analysis

GraphPad Prism software (version 9, San Diego, CA, USA) was used for statistical analysis. Statistical comparisons between vaccinated groups were performed *via* one way ANOVA with *post-hoc* Bonferroni multiple comparisons with an alpha of 0.05 (α ≤ 0.05). The nonparametric Kruskal-Wallis test was used to test differences between groups for the macroscopic and microscopic lesion scoring.

## Results

### NA2 VLPs Demonstrated Immunogenicity in the Swine Model

In previous work, we demonstrated that NA2 VLPs produced using a recombinant baculovirus (rBV) co-infection expression model in *Trichoplusia ni* insect cells contained enzymatically active NA suggestive of proper tertiary and quaternary protein structure (27). Furthermore, the NA2 VLPs were immunogenic in a murine model and protective in a lethal murine H3N2 challenge model (27). To examine the applicability of NA2 VLP vaccination in a swine model, we first investigated the immunogenicity of adjuvanted NA2 VLPs in prime-boost vaccination regimen in pigs. Three groups of pigs (n=5) were vaccinated in a prime-boost regimen 21 days apart at day 0 and day 21 with a NA2 VLPs, a commercial swine influenza A licensed quadrivalent whole inactivated virus vaccine (QWIV) (FluSure XP, Zoetis), or mock vaccinated with water-in-oil-in-water adjuvant only. Serum was collected on day 0 before prime vaccination, on day 21 at boost vaccination, on day 35, and on day 48. Anti-NA IgG titers were measured *via* ELISA.

**Figure 1:**
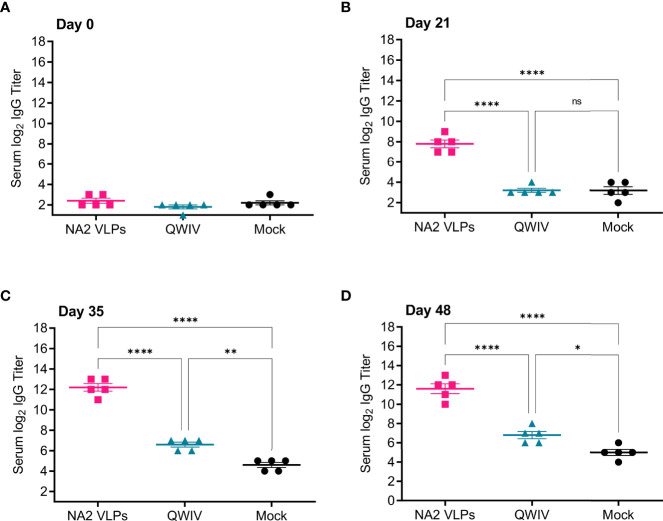
Vaccination with NA2 VLPS induces anti-NA serum IgG titers in swine. Three groups of pigs (n=5) were vaccinated in a prime-boost regimen 21 days apart at day 0 and day 21 with a commercial quadrivalent whole inactivated virus vaccine (QWIV), NA2 VLPs, or mock vaccinated with water-in-oil-in-water adjuvant only (Mock). Serum was collected on day 0 before prime vaccination, day 21 at boost vaccination, day 35, and at day 48. Anti-NA IgG titers were measured *via* ELISA. **(A)** Total anti-NA serum IgG titers at day 0 prior to prime vaccination. **(B)** Total anti-NA serum IgG titers at day 21 prior to boost vaccination. **(C)** Total anti-NA IgG titers two weeks post-boost vaccination on day 35. **(D)** Total anti-NA IgG titers prior to virus challenge with A/swine/NC/KH1552516/2016 on study day 48. Horizontal bars represent the mean IgG titer for each respective group. Statistical analysis was performed by one-way ANOVA test. * = p < 0.05; ** = p < 0.01; **** = p < 0.0001. ns = no statistical difference.

Prior to vaccination on day 0, all pigs were seronegative for NA specific IgG (**Figure 1A**). After prime vaccination on day 21, only pigs vaccinated with NA2 VLPs showed a measurable anti-NA IgG titer (**Figure 1B**). Interestingly, QWIV vaccinated pigs showed no anti-NA response above background after a single prime vaccination and, as expected, neither did the mock vaccinated group (**Figure 1B**). At 2 weeks post-boost vaccination, NA2 VLP vaccinated pigs demonstrated a statistically significant increase in NA specific serum IgG titers of approximately 50-fold compared to QWIV vaccinated pigs (p < 0.0001) (**Figure 1C**). QWIV vaccinated pigs also showed a mean increase of approximately 4-fold in NA specific IgG titers over mock vaccinated pigs at 2 weeks post-boost vaccination (p < 0.01) (**Figure 1C**). At four weeks post-boost, this trend continued with NA2 VLP vaccinated pigs maintaining an approximate 40-fold increase in anti-NA IgG titers compared to QWIV vaccinated pigs (p < 0.0001) and QWIV vaccinated pigs maintaining an approximate 3.5-fold increase in IgG titers over mock vaccinated pigs (p < 0.05) (**Figure 1D**).

These data suggest that NA2 VLPs are immunogenic and elicit high serum anti-NA IgG titers after vaccination. In addition, these data suggest that two doses of QWIV are required to elicit a modest but measurable anti-NA2 IgG response.

### NA2 VLPs Induced Functional Antibody Responses Directed at Neuraminidase

While anti-NA antibody responses have historically been recognized as important in small animal models and in human IAV infection (50–52), more recently the functional anti-NA antibody response capable of inhibiting NA enzymatic activity has been correlated with reduction of disease in humans (53, 54). Along these lines, we next measured the ability of sera from vaccinates to inhibit the NA enzymatic activity of the NA2 antigen contained in both the NA2 VLPs (A/Perth/16/2009) and the H3N2 challenge virus (A/swine/NC/KH1552516/2016).

**Figure 2:**
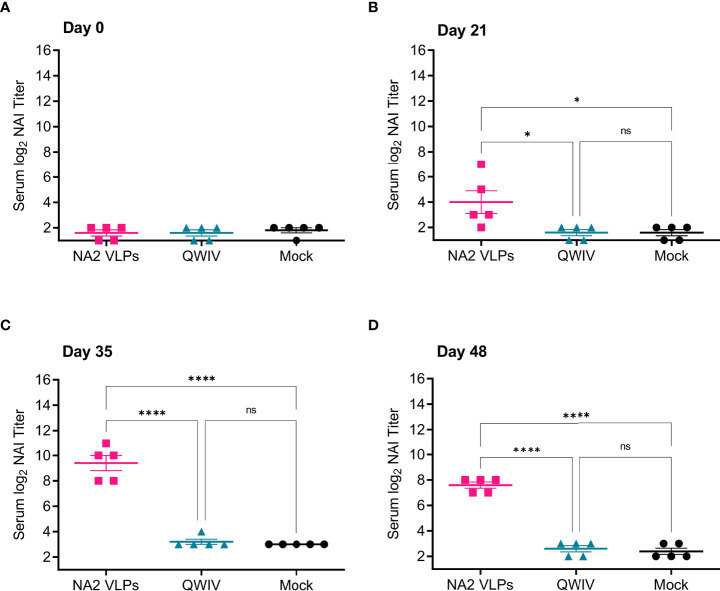
Vaccination with NA2 VLPS induces functional anti-NA antibodies capable of inhibiting A/Perth/16/2009 NA activity *in vitro*. Three groups of pigs (n=5) were vaccinated in a prime-boost regimen 21 days apart at day 0 and day 21 with a commercial QWIV, NA2 VLPs, or mock vaccinated with water-in-oil-in-water adjuvant only (Mock). Serum was collected on day 0 before prime vaccination, day 21 at boost vaccination, day 35, and at day 48. Neuraminidase inhibition (NAI) titers were defined as the reciprocal of the dilution inhibiting at least 50% of the NA activity of A/Perth/16/2009 in NA2 VLPs. **(A)** NAI titers at day 0 prior to prime vaccination. **(B)** NAI titers at day 21 prior to boost vaccination. **(C)** NAI titers two weeks post-boost vaccination on day 35. **(D)** NAI titers prior to virus challenge with A/swine/NC/KH1552516/2016 on study day 48. Horizontal bars represent the mean NAI titer for each respective group. Statistical analysis was performed by one-way ANOVA test. * = p < 0.05; **** = p < 0.0001; ns = no statistical difference.

We first evaluated the ability of sera collected from vaccinates to inhibit enzymatic activity the A/Perth/16/2009 NA2 antigen contained in the NA2 VLPs. Interestingly, while the QWIV vaccine induced a measurable anti-NA IgG response post-boost vaccination (**Figures 1C, D**), sera collected from WIV vaccinated animals did not show elevated neuraminidase inhibition (NAI) titers above mock vaccinated pigs at any timepoint (**Figures 2A–D**). As expected, sera from mock vaccinated animals also did not show any appreciable NAI titer at any timepoint sampled (**Figures 2A–D**). However, NA2 VLP vaccinated pigs showed a statistically significant elevation in NAI titer compared to mock and QWIV vaccinated animals after prime vaccination (p < 0.05) (**Figure 2B**). This trend continued post-boost vaccination at day 35, with sera from all NA2 VLP vaccinates showing elevated NAI titers compared to mock and QWIV vaccinates (p < 0.0001) (**Figure 2C**). Finally, sera collected at day 48 from NA2 VLP vaccinates also showed elevated NAI titers compared to mock and QWIV vaccinates (p < 0.0001) (**Figure 2D**).

**Figure 3:**
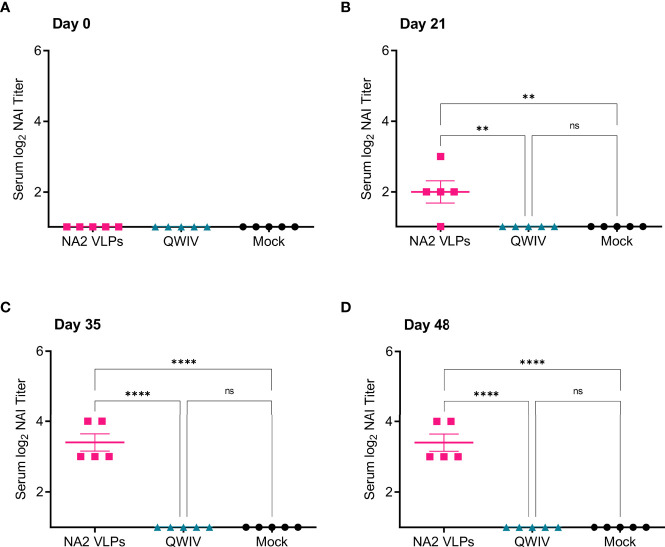
Vaccination with NA2 VLPS induces functional anti-NA antibodies capable of inhibiting A/swine/NC/KH1552516/2016 NA activity *in vitro*. Three groups of pigs (n=5) were vaccinated in a prime-boost regimen 21 days apart at day 0 and day 21 with a commercial QWIV, NA2 VLPs, or mock vaccinated with water-in-oil-in-water adjuvant only (Mock). Serum was collected on day 0 before prime vaccination, day 21 at boost vaccination, day 35, and at day 48. Neuraminidase inhibition (NAI) titers were defined as the reciprocal of the dilution inhibiting at least 50% of the NA activity of A/swine/NC/KH1552516/2016 virus. **(A)** NAI titers at day 0 prior to prime vaccination. **(B)** NAI titers at day 21 prior to boost vaccination. **(C)** NAI titers two weeks post-boost vaccination on day 35. **(D)** NAI titers prior to virus challenge with A/swine/NC/KH1552516/2016 on study day 48. Horizontal bars represent the mean NAI titer for each respective group. Statistical analysis was performed by one-way ANOVA test. ** = p < 0.01; **** = p < 0.0001; ns = no statistical difference.

Next, we investigated the ability of sera collected from vaccinates to inhibit the NA activity of the NA2 antigen of the H3N2 challenge virus A/swine/NC/KH1552516/2016. Similar to results obtained for the inhibition of the NA2 antigen used in the VLP vaccine, A/Perth/16/2009 (**Figure 2**), at no timepoint post-prime or boost vaccination did sera from mock vaccinated or QWIV vaccinated pigs demonstrate the ability to inhibit the NA activity of the challenge virus (**Figures 3A–D**). However, sera collected from NA2 VLP vaccinated animals demonstrated significantly elevated NAI titers compared to both mock and QWIV vaccinated groups post-prime vaccination (p < 0.01) (**Figure 3B**). As expected, this trend continued post-boost vaccination with NAI titers of sera from NA2 VLP vaccinates showing a significant increase compared to sera from QWIV and mock vaccinated groups (p < 0.001) (**Figures 3C, D**).

Appreciable NAI titers were observed in NA2 VLP vaccinated pigs both post-prime and post-boost vaccination against both antigens assayed, suggesting that NA2 VLP vaccination induced a broad anti-NA functional antibody response. Conversely, vaccination with QWIV did not significantly differ in ability to inhibit NA activity compared to mock vaccination at any timepoint against either antigen, suggesting that QWIV vaccination either does not induce functional anti-NA antibody capable of inhibiting NA enzymatic activity or does so at a level below the limit of detection against NA2 antigen from A/Perth/16/2009 or A/swine/NC/KH1552516/2016.

### Vaccination With QWIV Vaccine Induced HAI Antibodies Above Protective Levels After Second Vaccine Dose

As vaccination with QWIV did not induce functional antibodies capable inhibiting NA enzymatic activity in the form of measurable NAI titers, we next investigated the ability of vaccination with QWIV to induce antibodies capable of inhibiting hemagglutination. While the FDA and EMA consider serum hemagglutination inhibiting (HAI) titers greater that 1:40 to be a correlate of protection in humans (53), no such specifically quantified HAI titer correlated to protection against disease exists in swine. An HAI assay was performed for the assessment of HA-specific serum antibodies induced by the QWIV vaccine. Serum samples collected at day 0, 21, 35 and 48 were analyzed by HAI assay to investigate the magnitude of functional anti-HA antibody levels against the heterologous A/SW/NC/KH1552516/2016 challenge virus. As expected, sera from NA2 VLP and mock vaccinated animals did not show a measurable HAI titer at any timepoint assayed (**Supplementary Figure 1**). QWIV vaccinated animals demonstrated elevated mean HAI titers 2 weeks post-boost vaccination (80 ± 48.99) and four weeks post-boost vaccination (96 ± 60.66) (**Supplementary Figure 1**). Interestingly, while QWIV vaccinated animals had detectable HAI titers post-boost vaccination, all QWIV vaccinated individuals had an HAI titer below the limit of detection for the assay (1:20) after a single prime vaccination.

As suspected, NA2 VLP and mock vaccinated pigs did not demonstrate measurable HAI titers at any point post vaccination. However, QWIV vaccination induced appreciable HAI titers post-boost vaccination. While there is no reported HAI titer correlating with protection against IAV disease in swine, the human HAI correlate of protection may highlight the necessity of a second immunization with QWIV vaccine to elicit robust anti-HA functional antibodies in naïve pigs.

### NA2 VLP Vaccination Resulted in the Generation of Robust Virus-Specific Cell-Mediated Responses

Cell-mediated immunity (CMI) plays an important role in the host immune response in conferring protection against virus-related illness and in the establishment of long-term immunological memory (55). The presence of pre-existing T cell immunity in humans is critical for viral clearance from influenza infected cells (56, 57) and recovery from disease (38, 58). In the mouse model, T cells correlate with reduced virus shedding even when antibodies are absent (59–62). Studies analyzing in depth T cell immunity in response to immunization or infection against influenza virus in pigs are very limited (63–65). There is evidence that some CMI responses occur in lungs of pigs infected with swine influenza viruses (65–67) but these responses do not prevent infection (68). Talker et al. reported that at 44 days post-infection the IAV specific response comprised primarily IFN-γ single producing cells in the lung, TNF-α single producers in the tracheobronchial lymph nodes (TBLN) and triple producers (IFN-γ, TNF-α and IL-2) in the blood (47, 63).

**Figure 4:**
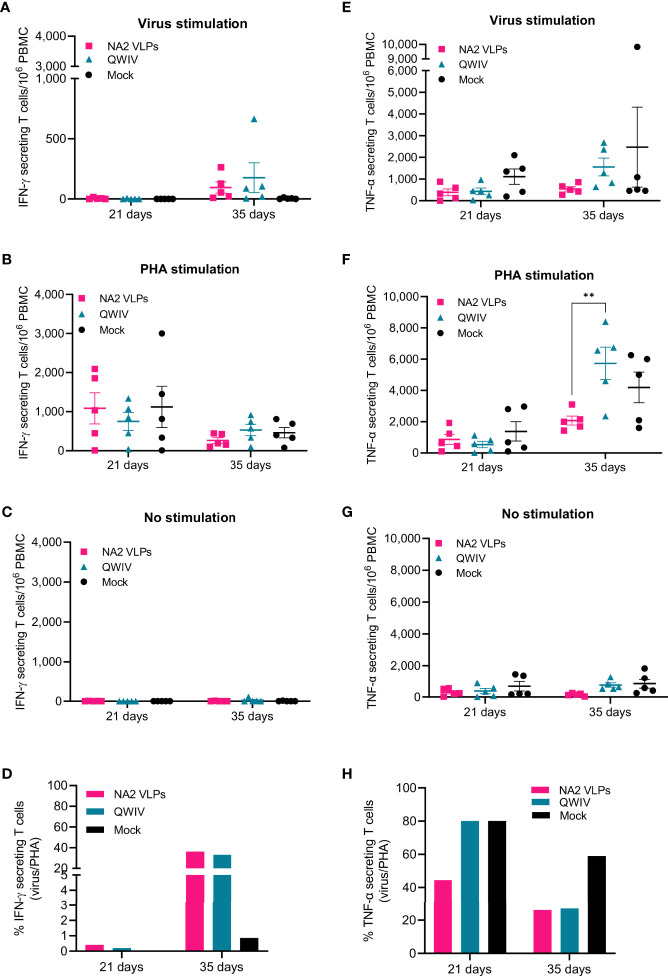
Vaccination with NA2 VLPS decreases TNF-α secreting T cells compared to mock vaccination following infection with A/swine/NC/KH1552516/2016 virus. PBMCs from pigs vaccinated in a prime-boost regimen 21 days apart with a QWIV, NA2 VLPs, or mock vaccinated with water-in-oil-in-water adjuvant were collected at 21 and 35 days and re-stimulated with A/swine/NC/KH1552516/2016 virus or PHA for enumeration of cytokine secreting cells. They were tested for IFN-γ secreting cells following **(A)** virus re-stimulation. **(B)** PHA re-stimulation. **(C)** no stimulation **(D)** ratio of virus vs PHA stimulated cells They were tested for TNF-α secreting cells following **(E)** virus re-stimulation. **(F)** PHA re-stimulation. **(G)** no stimulation **(H)** ratio of virus vs PHA stimulated cells. Pink squares represent NA VLP vaccinated pigs, cyan triangkes represent commercial quadrivalent whole inactivated virus vaccinated pigs (QWIV), and black circles represent mock vaccinated pigs. Horizontal lines represent the mean for each group. Statistical analysis was performed by two-way ANOVA test. Error bars represent SEM. ** = p < 0.01.

To determine whether pig immunization with NA2 VLPs leads to increased generation of NA2-specific activated T cells, we enumerated vaccine-specific IFN-γ and TNF-α producing T cells at 21 days post-prime and 14 days post-boost (day 35) and compared them to the QWIV vaccinated group following A/swine/NC/KH1552516/2016 virus or PHA stimulation. A single vaccine dose did not increase the IFN-γ secreting cells (**Figures 4A, B**). At two weeks post-boost though, we observed 2-fold differences between QWIV and NA2 VLP vaccinated pigs in virus-specific or PHA stimulated PBMCs despite the lack of significant statistics, that resulted in similar ratios of virus specific vs polyclonally stimulated cells (**Figure 4D**). Swine virus and PHA stimulation increased at similar levels the TNF-α secreting cell numbers of NA2 VLP and QWIV vaccinated pigs, post-prime (**Figures 4E, F**), whereas booster vaccination led to 4-fold increase of TNF-α secreting cells in the QWIV group with no changes in the NA2 VLP group following virus stimulation. At the same time point we observed 10-fold and 2.5-fold increases in PHA stimulated PBMCs from the QWIV and NA2 VLP groups respectively (p=0.002). These changes resulted in similar ratios of virus specific vs polyclonally stimulated cells (V/P ratio) for both vaccinated groups whereas the mock vaccinated pigs showed 2-fold higher ratio in suggesting similar effects of NA2 VLP and QWIV vaccine effect on cell mediated immunity (**Figure 4H**).

### Challenge With A/Swine/NC/KH1552516/2016 did not Result in Clinical Disease in Vaccinated Animals

A scoring system was applied for the evaluation of clinical disease after challenge with A/Swine/NC/KH1552516/2016. Recorded scores were used for monitoring the clinical status of the animals and for extrapolation of vaccine induced protection. From day -2 prior to challenge through day 5 post-infection, rectal temperature, respiratory rate, and disease signs, in the form of presence of coughing or weakness/depression, were recorded daily. The score range of clinical disease varied from 0 to 8, with 8 indicating the most severe clinical disease. Throughout the observation period, no statistically significant differences were observed between the three groups of pigs (**Supplementary Figure 2**). Overall, intranasal challenge resulted in mild clinical disease manifestation. This was not unexpected, as an intratracheal inoculation with a high dose of IAV is required to replicate severe respiratory disease in the swine model (69).

### Vaccination With NA2 VLPs Decreased Pulmonary Neutrophilic Infiltration Similarly to QWIV Group

**Figure 5:**
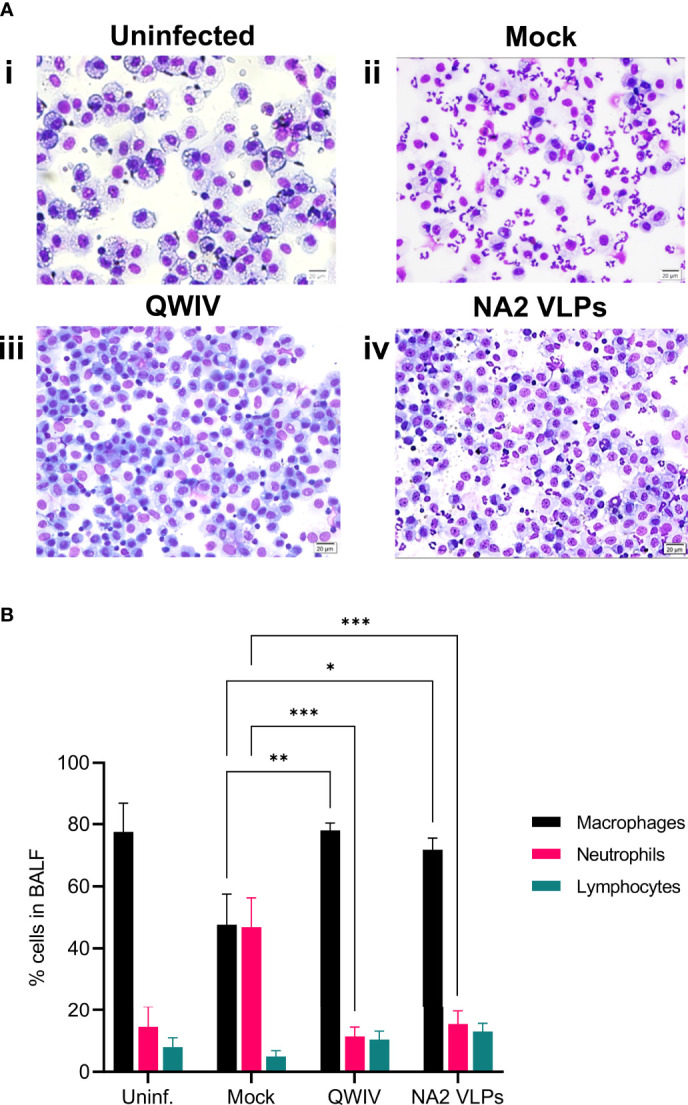
Vaccination with NA2 VLPs and swine QWIV vaccine reduces neutrophilic infiltration in bronchoalveolar lavage fluid collected from pig lungs post-infection. Bronchoalveolar lavage fluid (BALF) was collected from lungs at necropsy 5 days post-infection. **(A)** Separated cells were added to slides and stained with Wright-Giemsa stain and differential cell counts were determined microscopically (i) uninfected unvaccinated group (ii) mock vaccinated group (iii) QWIV vaccine group (iv) NA2 VLPs vaccine group (20 μm scale bar, 40x magnification) **(B)** Cell counts were expressed as a percentage of total cells. Black bars represent the mean percentage of macrophages, pink bars the mean percentage of neutrophils, and cyan bars represent the mean percentage of lymphocytes in each experimental group. Error bars represent the Standard Error of Mean (SEM). Statistical analysis was performed by two-way ANOVA test. * = p < 0.05; ** = p < 0.01; *** = p < 0.001.

To assess pulmonary infiltration of inflammatory cells, which in swine corelates with the development or prevention of respiratory disease (70), bronchoalveolar lavage fluid (BALF) was collected from all animals at necropsy 5 days post-infection. In healthy uninfected pigs (n=2), the mean ratio between the cellular elements in BALF was 77.5% resident macrophages, 14.5% small sized lymphocytes and 8% non-degenerate neutrophilic granulocytes (**Figures 5Ai**, **8B**). The unvaccinated control animals exhibited statistically significant higher percentages of neutrophils (46.8 ± 21.16) and lower percentage of macrophages (47.6 ± 22.11) compared to the vaccinated groups (**Figures 5Aii**, **8B**). These findings support the presence of moderate to severe pulmonary neutrophilic inflammation, typical of uncomplicated influenza virus infection. In contrast to the neutrophilic accumulation observed in the BALF samples of unvaccinated pigs, the majority of BAL cells were macrophages in vaccinated animals (78 ± 5.339 and 71.8 ± 8.389, QWIV and NA2 VLP vaccine group, respectively) with the presence of low percentage of neutrophils (**Figures 5Aiii, iv**, **8B**). The QWIV and NA2 VLP vaccinated animals showed significant reduction (p<0.0001 and p=0.0003, respectively) in lung neutrophilic infiltration (11.4 ± 6.693 and 15.4 ± 9.45, respectively) compared to unvaccinated pigs. No significant differences were identified in the percentage of lymphocytes between the groups. These data suggest that vaccination of pigs with NA2 VLPs or QWIV vaccine reduces neutrophilic infiltration after IAV infection.

### Vaccination With NA2 VLPs Reduced Viral Replication in the Lower Respiratory Tract of Infected Swine

**Figure 6:**
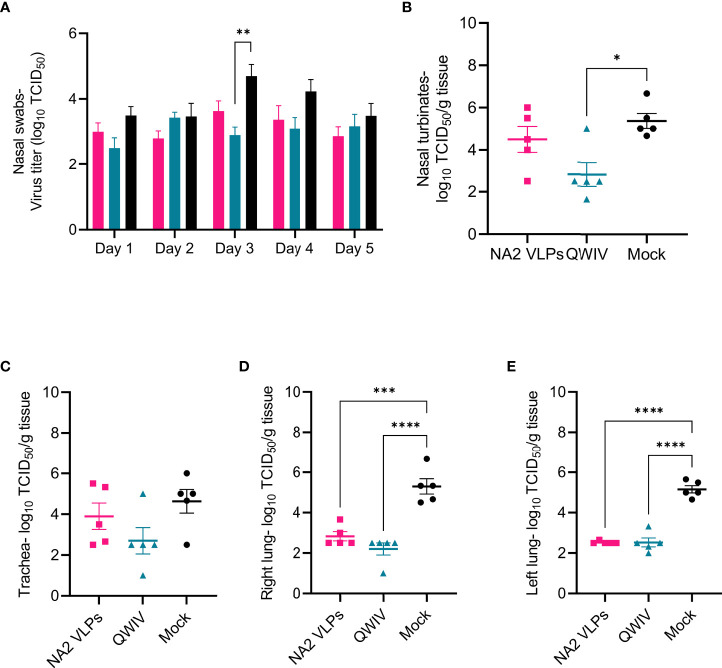
Vaccination with NA2 VLPs and the commercially available swine QWIV vaccine reduces viral replication in pig lungs post-infection. Nasal swabs were collected from pigs daily post-challenge through necropsy on day 5 and virus titers were measured *via* TCID_50_ assay. Tissue samples from the respiratory tract including nasal turbinates, trachea, right lung lobes including the accessory lung lobe, and left lung lobes were harvested 5 days post-infection at necropsy. Following tissue homogenization, virus replication was measured in each respiratory compartment *via* TCID_50_ assay of tissue homogenates. **(A)** Viral titers from nasal swabs **(B)** Mean viral titers of homogenized nasal turbinates. **(C)** Mean viral titers of homogenized trachea tissue. **(D)** Mean viral titers of right lung lobe tissue homogenates. **(E)** Mean viral titers of left lung lobe tissue homogenates. Pink squares represent NA VLP vaccinated pigs, cyan triangles represent commercial QWIV immunized pigs, and black circles represent mock vaccinated pigs. Horizontal lines represent the mean for each group. Error bars represent SEM. Statistical analysis was performed by two-way ANOVA test **(A)** and one-way ANOVA test **(B–E)**. * = p < 0.05; **= p < 0.01; *** = p < 0.001; **** = p < 0.0001.

To evaluate virus shedding post-challenge, nasal swabs were collected daily through day 5 and virus was measured *via* titration in a TCID_50_ assay. Overall, differences observed in mean virus titer collected from nasal swabs post-challenge were not statistically significant between the three vaccinated groups on days 1, 2, 4, and 5 post-infection (**Figure 6A**). However, samples on day 3 post-infection demonstrated a significant reduction of greater than one-log of virus was observed in nasal swabs collected from QWIV vaccinated pigs compared to mock vaccinated pigs (p=0.0099). Although the difference wan not statistically significant, mean viral shedding recovered from nasal swabs trended lower in the NA2 VLP and QWIV vaccinated animals compared to mock vaccinated pigs.

Next, we investigated the ability of vaccination to inhibit viral replication throughout the respiratory tract. Tissue samples were collected at necropsy, homogenized, and viral titers measured *via* TCID_50_ assay. Pigs vaccinated with the QWIV vaccine demonstrated a statistically significant reduction in viral load in homogenized nasal turbinates compared to the unvaccinated animals (p=0.014) but not compared to NA2 VLP vaccinated pigs (**Figure 6B**). Vaccination with NA2 VLPs did not significantly affect virus replication in nasal turbinates relative to mock vaccination. Additionally, mean tracheal viral loads appeared to be reduced in pigs vaccinated with either the QWIV vaccine or NA2 VLPs compared to mock vaccinated pigs, although the reduction was not significant (**Figure 6C**). In contrast to the upper respiratory tract where no clear differences were observed between our experimental vaccine and the mock group, in the lower respiratory tract (right and left lung) both vaccination with NA2 VLPs and the QWIV vaccine performed in a similar fashion resulting in statistically significant reduction of viral replication (p < 0.0001) (**Figures 6D, E**). In summary, vaccination of pigs with both NA2 VLPs and QWIV vaccine reduced viral replication after challenge with A/swine/NC/KH1552516/2016.

### Low Pulmonary Histopathology Scores Were Indistinguishable Between NA2 VLP and QWIV Vaccinated Groups Post-Challenge

**Figure 7:**
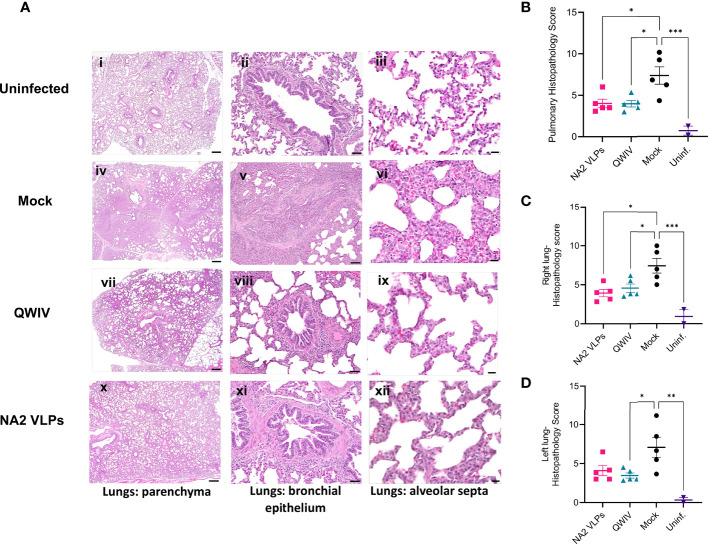
Vaccination with NA2 VLPs and the commercially available swine QWIV reduces total histopathology scores in pig lungs post-infection. Lung tissues were collected at necropsy 5 days post-infection and fixed in formalin. Five µm sections were stained with H&E and examined by light microscopy. Lung sections were scored by a board-certified veterinary pathologist. **(A)** H&E-stained representative lung tissue sections from each respective experimental group. Parenchyma 500 μm scale bar (2x magnification), bronchial epithelium 50 μm scale bar (20x magnification); alveolar septa 20 μm scale bar (40x magnification). **(B)** Mean lung histopathology scores from each vaccinated group. **(C)** Right lung mean histopathology scores. **(D)** Left lung mean histopathology scores. Pink Squares represent the NA2 VLP vaccinated pigs, cyan triangles the QWIV vaccinated pigs, black circles the mock vaccinated pigs and magenta triangles healthy uninfected pigs. Error bars represent SEM. Statistical analysis was performed by one-way ANOVA test. * = p < 0.05; ** = p < 0.01; *** = p < 0.001.

Gross pulmonary pathology was evaluated for the assessment of macroscopic lung lesion profiles. The majority of unvaccinated animals displayed dark red, multilobular to coalescing consolidation of the cranioventral regions of lungs consistent of uncomplicated influenza virus infection in swine (**Figures 7A iv–vi**). The macroscopic lung lesions and overall score of QWIV vaccine group (**Figures 7A vii–ix**) were indistinguishable from those in the NA2 VLP group (**Figures 7A x–xii**), and although the scores were lower compared to the unvaccinated control group, there was no statistically significant difference between the groups Next, the severity of microscopic lesions in the lung lobes, trachea, and nasal turbinates were evaluated. The composite pulmonary histopathology score was significantly higher in the unvaccinated control animals (7.36 ± 2.35) compared to the NA2 VLP (4.01 ± 1.15, p=0.02) and QWIV vaccine group (3.98 ± 0.87, p=0.019) (**Figures 7B–D**). In particular, the unvaccinated control group demonstrated higher severity lesions regarding the interstitial thickening of the alveolar septa and lymphocytic peribronchiolar cuffing compared to the animals in the NA2 VLP vaccinated and QWIV vaccine group. However, no statistically significant differences were observed in the microscopic nasal turbinate and trachea lesion scores, between vaccinated and unvaccinated control animals (**Supplementary Figure 3**). These data demonstrate that vaccination of pigs with NA2 VLPs or QWIV reduces both macroscopic and microscopic pulmonary lesions while reducing lung histopathology scores.

### NA2 VLP Vaccination Reduces Lung Inflammation Following Exposure to Influenza Virus

**Figure 8:**
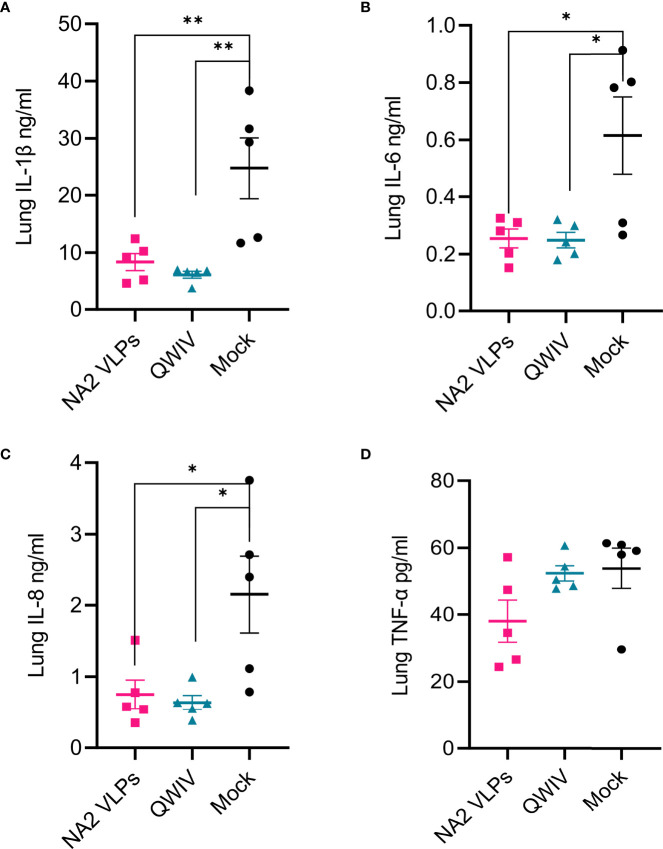
Vaccination with NA2 VLPs reduced lung inflammation in pig lungs post-infection. Lung homogenates prepared from tissues collected at necropsy of animals vaccinated with QWIV, NA2 VLPs or adjuvant alone 5 days post-infection, were tested for cytokine and chemokine secretion with MILLIPEX. **(A)** IL-1β **(B)** IL-6 **(C)** IL-8 **(D)** TNF-α. Pink squares represent NA2 VLP vaccinated pigs, cyan triangles QWIV vaccinated pigs, and black circles mock vaccinated pigs. Error bars represent SEM. Statistical analysis was performed by one-way ANOVA test. * = p < 0.05; ** = p < 0.01.

H1N1 infection induces the transcription of genes encoding cytokines such as IL-1β, IL-6, IL-8 and IL-12A, TNF-α in endothelial cells and lung fibroblasts and IL-1β expression increases the inflammation of lung cells (71). Clinical studies in children infected byH1N1 virus demonstrated an early and significant upregulation of IL-1β and IL-6 plasma expressions indicating a proinflammatory role that contributes to airway inflammation and bronchial hyperactivity (72). Interleukin-6 though has a pleiotropic nature leading to the upregulation of T cells and IgG isotype switching, inflammatory resolution, tissue remodeling and lung repair, migration and phagocytic activity of macrophages, prevention of viral-induced apoptosis in lung epithelial cells (73, 74). Van Reeth et al. reported that swine influenza virus induced acute respiratory disease and lung damage by itself and that the outcome of the infection was tightly associated with the production of IFN-alpha, TNF-alpha, IL-1 and IL-6. In challenge studies of SIV-vaccinated pigs, levels of IFN-alpha, TNF-α and IL-6, but not IL-1 were correlated with clinical and virological protection (75). Lung lysates taken from NA2 VLP and QWIV vaccinated swine 5 days after infection with A/swine/NC/KH1552516/2016 and tested for 13 major Th1, Th2 and inflammatory cytokines, showed a statistically significant 3-fold increase of IL-1β (p=0.007 and 0.003) (**Figure 8A**), IL-6 (p=0.016 and 0.015) (**Figure 8B**) and IL-8 (p=0.023 and 0.015) (**Figure 8C**) in the infected mock vaccinated group as compared to challenged swine that had received NA2 VLP or QWIV vaccine. Interestingly, we did not see statistical differences in TNF-α levels between vaccinated swine following viral challenge despite an approximately 30% reduction in the NA2 VLP group (**Figure 8D**).

## Discussion

While vaccine development has historically focused on anti-HA responses (10), anti-NA responses have recently been identified as a stronger correlate of protection against disease caused by IAV in humans. Although VLPs containing only the NA1 surface glycoprotein have been previously studied in small animal models, only one study to-date has investigated VLPs containing only the NA2 surface glycoprotein in a small animal model. Additionally, only one study to-date has investigated VLPs containing HA and NA produced in insect cells in a swine model. Here, we investigated the immunogenicity and efficacy of NA2 VLPs in an H3N2 infection model. Separating the relative contributions of anti-HA and anti-NA immunity to overall vaccine efficacy is difficult using traditional whole inactivated virus or split virus vaccines, and NA VLPs provide a useful tool to examine the contribution of NA specific immunity afforded through vaccination. In the current study, vaccination with NA2 VLPs offered similar protection against clinical signs of IAV disease, reduction in viral replication in lung tissues, and reduction in lung inflammation to a licensed, commercially available QWIV vaccine.

Interestingly, while vaccination with QWIV resulted in robust functional anti-HA immunity, capable of inhibiting hemagglutination after boost vaccination (**Supplementary Figure 1**), QWIV vaccination barely induced a measurable anti-NA2 response and no measurable functional anti-NA2 response. Similarly, previous work evaluating HA and NA immune responses in mice concluded that the presence of both HA and NA on the same inactivated virion led to antigenic competition, with HA being immunodominant over NA in priming B cells and T cells (76). However, subsequent work demonstrated that detergent disruption and disassociation of HA and NA from the same inactivated virion eliminated this antigenic competition (77, 78). Along these same lines, more recent work has suggested that extending the length of the NA stalk by 30 amino acids augments anti-NA responses in a murine vaccination model with whole inactivated virus without compromising anti-HA responses (79). Further research is needed to determine if such an approach would augment anti-NA responses in a swine model.

While vaccination with NA2 VLPs resulted in an equivalent reduction in inflammation and disease compared to QWIV vaccination, the equivalent reduction in viral replication in swine lungs compared to mock vaccinated pigs was notable. However, with respect to virus shedding, while mean virus titers recovered from nasal swabs were consistently lower in NA2 VLP vaccinated pigs, this difference was not significant through daily sample collection up to day 5 (**Figure 6**). Reduced viral mobility and subsequent viral transmission have been proposed as potential benefits of robust anti-NA functional immunity (2), however the absence of decreased viral shedding in NA2 VLP vaccinates does not support this proposal. Additional transmission studies employing cohoused naïve contact pigs could provide additional information on the effect of anti-NA immunity in virus dissemination and spread.

Another factor that has to be taken into account regarding nasal shedding is that intramuscular vaccination predominantly induces serum antibodies and to a lesser degree mucosal antibodies (80). While serum IgG antibodies can rapidly transudate through alveolar spaces and facilitate protection in pulmonary tissues, this process is generally impeded in the nasal mucosa due to the presence of a thicker mucosal layer (81). The major isotype that mediates nasal immunity is secretory IgA (82). In accordance with this, we presume that the intramuscular administered IAV vaccines examined in our report primarily conferred lung immunity, resulting in significant reduction of pulmonary virus replication. While we did not examine in this study the extent of mucosal immunity elicited by our experimental NA VP or the commercial swine IAV vaccine, the non-significant reduction of nasal shedding could be correlated with a less than robust stimulation and activation of nasal secretory IgA. Future work is needed to determine the role of intranasal administration of our NA2 VLPs construct in the stimulation of mucosal immunity and reduction of virus shedding.

A main objective of broadly protective IAV vaccine strategies is the induction of CMI. Cell mediated responses are not only associated with effective viral clearance after IAV infection but are also involved in protection against heterologous viruses, considering that they target conserved epitopes present on viral proteins (83, 84). Previous studies have demonstrated that unlike naïve T cells, primed memory CD4 T cells secrete IFN-γ, as well as other antiviral cytokines (IL-6, IL-12, CXCL9 and CXCL10), rapidly after IAV infection (85, 86). While the mechanism is not completely understood, these Th1-type responses producing IFN-γ, during the initial stages of infection, facilitate effective early viral clearance primarily through the activation and transmigration of CTL (Cytotoxic T Lymphocytes) and macrophages to the site of inflammation (87, 88). Consistent with this is the general notion that the presence of Th1 polarized IFN-γ secreting memory T cells is strongly correlated with positive clinical outcome after IAV infection. In our study, both NA2 VLP and commercial swine IAV elicited adequate, although with not significant differences between these groups, levels of IFN-γ secreting T cells after heterologous virus stimulation, in PBMCs collected at 2 weeks after boost vaccination, suggesting a competent Th1 response following infection with the heterologous A/SW/NC/KH1552516/2016 challenge virus.

In addition to IFN-γ, we measured TNF-α secreting cells in PBMCs collected post-prime and post-boost. TNF-α is a proinflammatory cytokine associated with systemic inflammation during the acute phase of IAV infection (89, 90). TNF-α promotes increased vascular permeability and extensive recruitment of inflammatory cells to the site of inflammation (91–93). These activities can contribute to lung tissue pathology due to vascular injury and destruction of the lung parenchymal cells (94, 95). Previous studies have demonstrated that while TNF-α can induce hyper-inflammatory responses that result in severe clinical outcome and lung tissue injury, this cytokine may have a limited impact in reducing virus replication and overall in virus clearance (91, 96–98). Interestingly, in our study we observed a two-fold higher ratio of virus specific vs polyclonally stimulated TNF-α secreting cells, in PBMCs collected from naïve mock vaccinated animals and QWIV vaccinated pigs, compared to PBMCs harvested from NA2 VLPs vaccinated animals post-prime vaccination. Additionally, we detected post-boost a two-fold decrease in the V/P ratio of TNF-α secreting T cells, in both NA2 VLPs and QWIV vaccinated pigs compared to mock vaccinated pigs. The ELISPOT results demonstrated that a single NA2 VLPs vaccination or two doses of QWIV vaccine were required to prime the pigs towards a sufficient, however less extensive, production of TNF-α by memory T cells compared to naïve T cells following virus stimulation.

While cell-mediated responses and particularly Th1 activation are required for effective virus clearance, a critical correlate of vaccine induced protection, is the elicitation of balanced pulmonary cytokine responses during IAV infection. Excessive production of proinflammatory cytokines, in the acute stage of viral infection, is associated with immunopathology and lung tissue injury (99, 100). In this report, the naïve mock vaccinated group demonstrated at necropsy elevated neutrophil levels in BALF, enhanced microscopic pneumonia and proinflammatory lung cytokine profile, including IL-1β, IL-6 and IL-8, compared to both NA2 VLPs and QWIV group of animals. Previous studies have demonstrated that aberrant production of IL-1β and IL-6 is associated with exacerbation of inflammatory response and immunopathology (101–103). In this respect, marked reduction of lung IL-1β and IL-6 levels may have contributed to the prevention of development of severe pulmonary lesions in the NA2 VLPs group. Interestingly the effect of IL-8 upregulation was impressively demonstrated in the neutrophilic recruitment in the lungs of mock vaccinated pigs after challenge (104). We detected a 4-fold increase of neutrophils post-infection compared to the NA2 VLP and QWIV groups (p=0.0001 and 0.0007 respectively) and a concomitant 40% reduction of macrophages (p=0.0011 and 0.011 respectively), resulting in equal representation of these populations in the BAL fluid (**Figure 5B**). In contrast, macrophages were the overwhelming majority in the QWIV (ratio 7:1) and NA2 VLP (ratio 5:1) vaccinated pigs similarly to the unvaccinated/uninfected control group (ratio 5:1). These findings suggest a strong anti-inflammatory role of vaccination by suppressing the chemotactic effect of IL-8.

In summary, immunization with a novel NA2 VLP vaccine was successful in significantly reducing virus replication, mitigating clinical disease, and controlling inflammation measured *via* neutrophilic infiltration, and pulmonary histopathology scoring in the porcine model. These findings contribute to the better understanding of anti-NA immunity in preventing IAV infection and disease in swine, as an animal model for studying influenza in humans, but also in swine production, since swine influenza is an economically important pathogen in the pork industry (105).

## Data Availability Statement

The raw data supporting the conclusions of this article will be made available by the authors, without undue reservation.

## Ethics Statement

The animal study was reviewed and approved by Auburn University Institutional Animal Care and Use Committee (IACUC).

## Author Contributions

VP, ZM, IS and CK designed the research. VP, ZM, VA, JY, PN, JN, DW and CK performed research. JY and KH performed histopathological analyses. MN performed BL cytology analyses. JJ performed ELISpot analyses, VP, ZM, IS and CK wrote the paper. All authors contributed to the article and approved the submitted version.

## Funding

This work was funded by the NIH/NIAID Centers of Excellence for Influenza Research and Surveillance (CEIRS) contract HHSN272201400004C and the Auburn University Alabama Agricultural Experiment Station (AAES). Virginia Aida is funded through the United States Department of Agriculture (USDA) Animal and Plant Health Inspection Service’s (APHIS) National Bio and Agro-Defense Facility Scientist Training Program.

## Conflict of Interest

The authors declare that the research was conducted in the absence of any commercial or financial relationships that could be construed as a potential conflict of interest.

## Publisher’s Note

All claims expressed in this article are solely those of the authors and do not necessarily represent those of their affiliated organizations, or those of the publisher, the editors and the reviewers. Any product that may be evaluated in this article, or claim that may be made by its manufacturer, is not guaranteed or endorsed by the publisher.
