# Supplementary material for: A Novel Neuraminidase Virus-Like Particle Vaccine Offers Protection Against Heterologous H3N2 Influenza Virus Infection in the Porcine Model
Source: Front Immunol. 2022 Jul 7;13:915364. doi: 10.3389/fimmu.2022.915364 (PMC9300842; doi:10.3389/fimmu.2022.915364)
Supplement: Supplementary file 1 [file DataSheet_1.docx]

Suppl. Figure 1

Suppl. Figure 1: Prime boost vaccination with commercial QWIV induces serum antibody responses capable of inhibiting hemagglutination of A/swine/NC/KH1552516/2016. Serum samples collected at day 0, 21, 35 and 48 were assayed for hemagglutination inhibition (HAI) titers against the heterologous H3N2 (A/SW/NC/KH15/2016) challenge virus. Bars represent geometric mean HAI titers and errors bars 95% CI. Sera were collected from mock vaccinated animals (black), NA2 VLP vaccinated animals (pink), and QWIV vaccinated animals (cyan).

Suppl. Figure 2

Suppl. Figure 2: Swine clinical disease scores after challenge with A/swine/NC/KH1552516/2016**.** Clinical scores including rectal temperature, respiratory rate and assessment of clinical demeanor were recorded daily throughout the challenge. Specifically, rectal temperature score ranged from 0 to 3 (< 39.4°C = 0, 39.4-39.9°C = 1, 40-40.5°C = 2, > 40.6°C = 3) , respiratory rate per minute score ranged from 0 to 2 (20-40 = 0, 41-59 = 1, > 60 = 2) and clinical behavior score, based on coughing (absent = 0, present = 1) and depression (absent = 0, present = 2) ranged from 0 to 3. Black circles represent the mock vaccinated pigs (Mock) (n=5), pink squares the NA2 VLP vaccinated pigs (n=5) and cyan triangles the QWIV vaccinated pigs (n=5). Statistical analysis was performed by two-way ANOVA test. * = p < 0.05; ** = p < 0.01.

Suppl. Figure 3


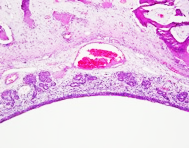

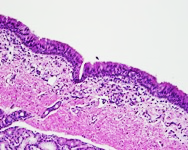

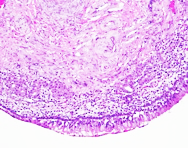

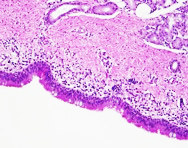

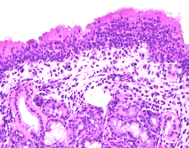

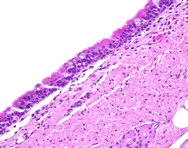

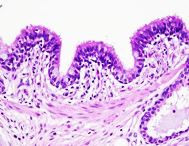

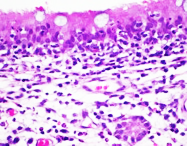


**Nasal Turbinates**

**Trachea**

**Mock infected**

**QWIV infected**

**Uninfected**

**NA2 VLPs infected**

**A**

**B**

Suppl. Figure 3: Vaccination with NA2 VLPs and the commercially available QWIV swine IAV vaccine did not affect total histopathology scores in pig nasal turbinates and trachea. Lung tissues were collected at necropsy 5 days post-infection and fixed in formalin. Five µm sections were stained with H&E and examined by light microscopy. Lung sections were scored by a board-certified veterinary pathologist. H&E-stained representative lung tissue sections from each respective experimental group in (A) nasal turbinates and (B) trachea.
